# Effect of Blood Flow Restriction on Gait and Mobility in Older Adults: A Systematic Review and Meta-Analysis

**DOI:** 10.3390/ijerph21101325

**Published:** 2024-10-07

**Authors:** Katherine L. Hsieh, Andrew Foster, Logan MacIntyre, Reagan Carr

**Affiliations:** Department of Physical Therapy, Byrdine F. Lewis College of Nursing and Health Professions, Georgia State University, Atlanta, GA 30303, USA; afoster67@student.gsu.edu (A.F.); lmacintyre2@student.gsu.edu (L.M.); rcarr14@student.gsu.edu (R.C.)

**Keywords:** aging, physical performance, walking, fall risk, occlusion training

## Abstract

Older adults demonstrate gait impairments that increase their risk for falls. These age-related mobility impairments are in part due to declines in muscle mass and strength. High-intensity exercise can improve muscle strength and mobility but may not be tolerable for older adults due to musculoskeletal injury and pain. Blood flow restriction (BFR) with lower-intensity exercise offers a strategy that may be more tolerable for older adults, but whether BFR improves gait and mobility in older adults is unclear. The purpose of this systematic review and meta-analysis was to determine the effect of BFR on gait and mobility in healthy older adults. PubMed, Embase, Cochrane Library, and CINAHL were systematically searched for articles utilizing BFR in older adults. Articles were included if adults were over 60 years, did not have chronic health conditions, had undergone randomized controlled trials, and presented objectively measured gait outcomes. The search identified 1501 studies, of which 9 were included in the systematic review and 8 studies in the meta-analysis. Outcome measures included the Timed Up and Go (TUG), 6-Minute Walk Test (6MWT), 400 m walk test, Short Physical Performance Battery (SPPB), and 10 m walk test. Meta-analyses found improvements in the TUG (mean difference (MD) = −0.71; 95% CI = −1.05, −0.37; *p* < 0.001) and SPPB (MD = −0.94; 95% CI = −1.48, −0.39; *p* < 0.001) in BFR compared to no BFR. There were no differences in gait speed (MD = 0.59; 95% CI = −0.22, 1.41; *p* = 0.16). BFR may be effective for gait and mobility tasks over shorter distances. Clinicians may consider incorporating BFR to improve mobility and gait function in older adults.

## 1. Introduction

Older adults aged 60 years and older make up 11% of the world’s population, and this number is expected to double to 2.1 billion by 2050 [1]. Due to physiological declines with aging, older adults demonstrate gait impairments that impact their ability to navigate in their communities [2]. For instance, older adults walk at slower speeds and with increased variability, which are associated with increased future falls [3,4]. Older adults also walk with shorter and wider strides and spend longer time in double limb support [5]. Part of these gait changes are due to declines in muscle strength, power, and endurance that occur with aging [6]. Because of these gait impairments, older adults are at risk for falls, disability, and frailty [2].

Resistance and aerobic exercise can improve neuromuscular function, mobility, and gait performance. Lower-extremity resistance training, for example, improves muscle strength and power that are needed for everyday activities such as walking up and down stairs or crossing over obstacles [7,8]. Jogging or biking increases cardiovascular capacity that is needed to walk in a city [9]. High-intensity exercise, however, is not always tolerable for older adults as it may lead to musculoskeletal injuries due to high mechanical stress [10]. Older adults may have contradictions (i.e., uncontrolled hypertension, diabetes) to performing heavy-load exercises that can increase their risk of cardiovascular events or joint pain [11]. Due to these limitations, offering safe and effective exercise for older adults is needed to improve their physical function and quality of life.

Blood flow restriction (BFR) offers an alternative method for high-load, high-intensity exercise while still increasing muscular strength and endurance [12,13]. BFR uses a pressurized cuff to restrict limb blood flow and oxygen supply [13]. In doing so, BFR creates muscle fatigue by recruiting high-threshold motor units when undergoing low-repetition and low-load exercises [13]. Previous studies have demonstrated that older adults utilizing BFR increased muscle strength and muscle hypertrophy [14,15]. Moreover, a systematic review and meta-analysis of 11 studies found that low-load BFR training increased muscle strength compared to low-load resistance training and produced similar increases in muscle mass compared to high-load resistance training [16]. Other studies have reported increases in muscle cross-sectional area and volume with BFR combined with walking and resistance training [17,18]. Several studies have also established the safety of using BFR with older adults [19,20].

While the evidence supports BFR to improve strength and hypertrophy in older adults, the evidence is less clear in regard to gait and mobility. Some studies have found improvements in physical function following BFR, such as with the 30-s chair stand or standing balance [21,22]. Studies more recently have examined walking function, but whether older adults will improve their gait following BFR is unclear. Using BFR to increase walking function can provide an alternative tool for older adults to maintain their independence and reduce their risk for frailty. Therefore, the purpose of this systematic review and meta-analysis was to determine the effect of BFR on gait and mobility in healthy older adults. We hypothesized that gait outcomes would improve for older adults exercising with BFR compared to without BFR.

## 2. Methods

### 2.1. Search Strategy

This systematic review and meta-analysis followed the guidelines provided in the PRISMA statement [23]. This study was not registered with the International Prospective Registry of Systematic Reviews. Multiple databases (PubMed, Embase, Cochrane, CINAHL) were searched for articles from inception until July 2024. Articles were searched for words containing “blood flow restriction”, “occlusion training”, “vascular occlusion”, “gait”, “walking”, “ambulation”, “gait speed”, “gait endurance”, “mobility”, “gait analysis”, “physical function”, “elderly”, “older adult”, “aging”, “aged”, “geriatric”, and other related key words. Three researchers (RC, AF, and LM) independently screened all articles by title and abstract. Articles meeting eligibility criteria were retrieved for full-text evaluation. Backward and forward reference searches of the included studies were performed to identify any other potential studies that met the eligibility criteria. Articles meeting the eligibility criteria following full-text evaluation were included in the systematic review. Any disagreements were resolved by further discussion with a fourth author (KH).

### 2.2. Eligibility Criteria

The PICOS framework based on the Participants, Interventions, Comparisons, Outcomes, and Study designs was adopted to define the inclusion and exclusion criteria [24]. Studies were included if (1) the average age of participants was 60 years or older, (2) the studies objectively measured gait as a primary or secondary outcome measure, and (3) the studies were randomized controlled trials. Studies were excluded if they (1) included participants with neurological disease (e.g., multiple sclerosis, spinal cord injury, Parkinson’s disease), chronic kidney disease, autoimmune disorders, or cancer; (2) included participants who were post-surgery or (3) were non-human; or (4) were non-English publications.

### 2.3. Data Extraction

Data from full-text articles were extracted for information, including (1) participant characteristics, (2) intervention design, (3) primary and secondary outcome measures, and (4) main results. In case of incomplete raw data availability, we contacted the corresponding author of the manuscript or extrapolated the data from figures if the authors could not be reached.

### 2.4. Statistical Analysis

For calculating the standardized mean difference (SMD), the difference in pre- and post-intervention mean and standard deviation values of gait measures for all groups in each study was used. The *I*^2^ statistics were used to determine statistical heterogeneity among the studies. A random-effects modeling approach was used when the pooled data had moderate (*I*^2^ = 50%−75%) or high heterogeneity (*I*^2^ > 75%) [25]. A fixed-effects modeling was used for low-heterogeneity comparison when *I*^2^ < 50% [25]. For each comparison, pooled effects sizes (ESs) were calculated with alpha set at 0.05. The effect treatment displayed as SMD was interpreted using the following classification: 0.2 represents a small effect, 0.5 a moderate effect, and 0.8 a large effect. Analyses were performed in SPSS version 29.

### 2.5. Quality Assessment & Quality of Evidence

The National Institutes of Health Quality Assessment Tool for randomized controlled trials was adopted to assess risk of bias for all studies [26]. Studies were assessed with 12 questions, with possible answers being yes, no, or cannot determine. The criteria that needed to be met were based on the following questions: (1) Was the research question clearly stated? (2) Were the inclusion and exclusion criteria of the study sample clearly stated? (3) Was the method of randomization adequate? (4) Were the main findings of the study clearly articulated? (5) Were groups similar at baseline on important characteristics that could affect outcomes? (6) Was gait or mobility measures well defined? (7) Were outcomes assessed using valid and reliable measures, implemented consistently across all study participants? (8) Was the protocol appropriate to measure gait or balance? (9) Was there high adherence to the intervention protocols for each treatment group? (10) Was the overall drop-out rate from the study at endpoint 20% or lower of the number allocated to treatment? (11) Was the sample size justified by a power analysis? And (12) Were potential confounders properly controlled for in the analysis? Overall quality was then assigned to each study as good, fair, or poor [26].

Study quality for the included studies was also assessed using the Cochrane Risk of Bias tool [27]. The tool includes a description and a judgment for each entry in a risk-of-bias table, wherein each entry addresses a specific feature of the study. The judgment for each entry involves a response of low risk of bias, high risk of bias, or unclear risk of bias, implying lack of information or uncertainty of potential for bias. Risk of bias was assessed according to the following domains: (1) random sequence generation; (2) allocation concealment; (3) blinding of participants and personnel; (4) blinding of outcome assessors; (5) incomplete outcome data; (6) selective outcome reporting; and (7) other potential bias (free of expertise bias). Risk of bias within each domain was assessed based on criteria provided by the *Cochrane Handbook* [27].

## 3. Results

### 3.1. Study Selection

**Figure 1:**
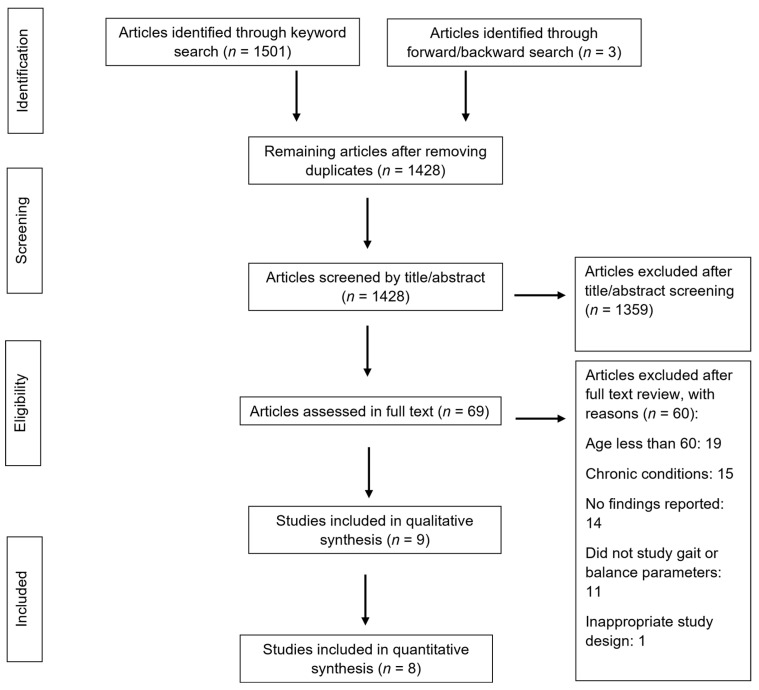
Study flow chart following the Preferred Reporting Items for Systematic Review and Meta-Analysis guidelines.

The initial search of databases yielded 1501 results. Three additional articles were identified through forward/backward search. After removing duplicates, 1428 articles were screened by title and abstract, and 69 articles were assessed in full text. Nine articles were included in the systematic review and eight articles in the meta-analysis [14,17,28,29,30,31,32,33,34]. The most common reasons for exclusion were including participants under 60 years of age (*n* = 19), including participants with chronic conditions (i.e., spinal cord injury, chronic kidney disease, multiple sclerosis) (*n* = 15), or not reporting findings (*n* = 14). Figure 1 depicts a flow diagram based on the Preferred Reporting Items for Systematic Review and Meta-Analysis guidelines.

### 3.2. Study Design and Characteristics

Study ID, sample size, intervention type, intervention length, cuff pressure, and main outcome measures are described in Table 1. A total of 240 participants were included in the systematic review and meta-analysis. Participants’ average age ranged from 62.9 to 72.3 years. Intervention length ranged from 6 to 16 weeks with a frequency between 1 and 3 times per week. Interventions incorporated BFR with walking, dual-task walking, or low-load resistance training. Cuffs were placed on the proximal part of the thigh or proximal portion of the leg for all studies. Control groups included walking, high-load resistance training, balance exercises, or usual activity. Resistance training exercises for all studies predominantly included lower limb exercises, such as leg extensions, leg press, and leg curls. Gait-related outcome measures included the Timed Up and Go (TUG), 6-Minute Walk Test (6MWT), 400 m walk test, 10 m walk test, and Short Physical Performance Battery (SPPB). The TUG examines dynamic balance, gait, and turning [35] while the 6MWT [36] and 400 m walk test [37] examine aerobic capacity and endurance. The SPPB includes three standing balance tasks, a 4 m walk test, and a five-times sit-to-stand that is scored from 0 to 12 with higher scores indicating greater physical function [38].

### 3.3. Methodological Quality

**Figure 2:**
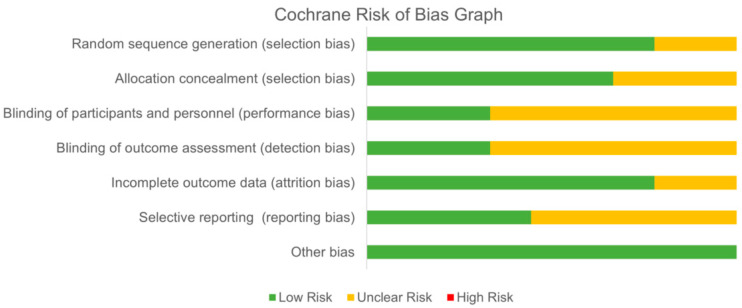
Risk of bias summary.

Study quality assessment for each study is depicted in Table 2. Seven of the studies were rated as good quality [14,28,29,30,31,32,34], and two of the studies were rated as fair quality [17,33]. The majority of the studies did not report a power analysis to justify their sample size calculation. It was also unclear for most studies if they adjusted for confounding variables in their analysis, and many did not report adherence rates to their interventions. The summary of quality evidence from the Cochrane Risk of Bias is depicted in Figure 2 with individual study evidence displayed in Table 3. Overall, there was low-to-unclear risk across all domains. Only one study had low bias across all domains [14]. Lowest risk was in selection bias and other bias. There was unclear risk in performance bias, detection bias, and reporting bias. Many studies did not report enough information to determine level of risk.

### 3.4. Effect of BFR on Timed up and Go

**Figure 3:**
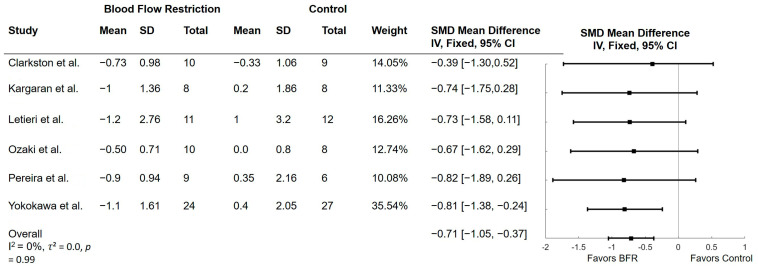
Forest plots of trials comparing blood flow restriction with no blood flow restriction on the Timed Up and Go. SD = standard deviation; SMD = standardized mean difference [14,17,29,32,33,34].

Seven studies examined the effect of BFR on the TUG. Three of the studies examined BFR with walking [17,28,29] while three examined BFR with low-intensity resistance training [14,33,34]. One study examined BFR with dual-tasking walking [32]. The results of these comparisons are shown in Figure 3. All studies found improvements in TUG time in the BFR groups compared to control. Time during the TUG significantly improved in BFR compared to control (mean difference (MD) = −0.71; 95% CI = −1.05, −0.37; *p* < 0.001). Heterogeneity was not significant with an *I*^2^ of 0% (*p* = 0.99) and *τ*^2^ of 0.0. One study was not included in the meta-analysis as SMD could not be calculated [28]. Results from Abe et al. [28] found that BFR with 20 min of walking improved TUG performance while those in the no exercise group did not improve TUG performance.

### 3.5. Effect of BFR on Gait Speed

**Figure 4:**
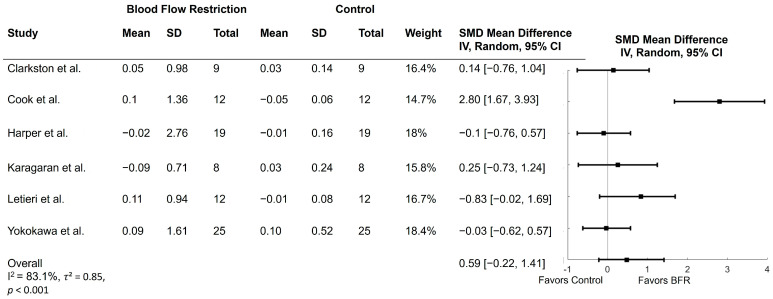
Forest plots of trials comparing blood flow restriction with no blood flow restriction on gait speed. SD = standard deviation; SMD = standardized mean difference [14,29,30,31,32,34].

Six studies examined the effect of BFR on gait speed. Gait speed was extracted from the 6MWT (*n* = 3) [14,29,32], 400 m walk test (*n* = 2) [30,31], and 10 m walk (*n* = 1) [34]. Five studies examined BFR with low-load resistance training on gait speed [30,31,32,34,39] while two studies examined BFR with walking on gait speed [29,32]. The results of these comparisons are shown in Figure 4. Four studies found improvements in gait speed in the BFR group compared to control [14,29,30,32], while two studies found no significant differences between groups [31,34]. Three of the studies that demonstrated increased gait speed utilized the 6MWT [14,29,32], and the other utilized the 400 m walk test [30]. Meta-analysis indicated that there were no significant differences between BFR and control for gait speed (MD = 0.59; 95% CI = −0.22,1.41; *p* = 0.16). Heterogeneity was significant with an *I*^2^ of 83.1% (*p* < 0.001) and *τ*^2^ of 0.85.

### 3.6. Effect of BFR on Short Physical Performance Battery

**Figure 5:**
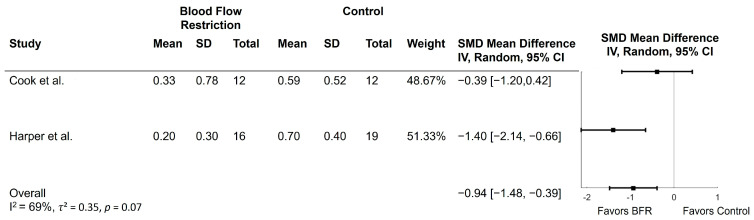
Forest plots of trials comparing blood flow restriction with no blood flow restriction on the Short Physical Performance Battery. SD = standard deviation; SMD = standardized mean difference [30,31].

Two studies examined the effect of BFR on the Short Physical Performance Battery [30,31]. Both studies compared low-load resistance training with BFR to high-load resistance training or flexibility exercises. The results of these comparisons are depicted in Figure 5. Scores on the SPPB improved in the BFR group compared to control (MD = −0.94; 95% CI = −1.48, −0.39; *p* < 0.001). Heterogeneity was moderate with an *I*^2^ of 69% (*p* = 0.07) and *τ*^2^ of 0.35. One study reported gait speed from the 4 m walk test within the SPPB, but the second study did not. Cook et al. [30] found that gait speed increased across the entire sample.

## 4. Discussion

The overall purpose of this study was to perform a systematic review and meta-analysis to determine whether BFR during exercise training improves gait and mobility compared to no BFR in older adults. After an initial search of 1501 studies, we identified 9 articles that met the eligibility criteria and 8 were included in the meta-analysis. Results from the meta-analysis found that performance on the TUG and SPPB improved following BFR intervention while there were no differences in gait speed. Average study quality assessment was good, and overall risk of bias was unclear or low.

The results partially supported our hypothesis that gait and mobility would improve from BFR training compared to no BFR. We found improvements in the TUG and SPPB, which both utilize leg strength to stand from and sit on a chair. The SPPB also includes static balance tasks that utilize ankle and hip strength to maintain postural control. BFR training in the included studies may have increased lower extremity muscle strength that led to improvements in the TUG and SPPB. BFR is also designed to promote hypoxia, increasing type II muscle fiber activation [12]. These adaptations may help improve gait speed over shorter distances rather than longer, endurance walking tasks. Gait speed, on the other hand, did not differ between groups. Five of the six studies that measured gait speed used the 400 m walk test or 6MWT to measure gait speed. BFR may not affect endurance or aerobic capacity that is needed for longer-distance walking tasks. Rather, BFR may have a greater effect on gait during shorter walking distances, such as with the TUG (3 m) and SPPB (4 m). BFR, when combined with low-intensity resistance training or walking, appears to have a greater effect on mobility when leg strength or power is utilized. Lastly, none of the studies included reported adverse events, supporting the safety of BFR use in an older adult population.

These changes in mobility may be due to multiple mechanisms from BFR. Occluding blood flow induces hypoxia, which may encourage motor recruitment of type II muscle fibers. Fast twitch muscle fibers generate greater force but fatigue faster [40]. In addition, BFR may also increase metabolic stress that increases lactate accumulation, stimulating growth hormone secretion and inducing muscle growth [41]. BFR may also cause intramuscular signaling pathways, such as mTORC 1 production, that increase protein synthesis [42]. These mechanisms may result in increased muscle strength and hypertrophy, which can assist with tasks associated with the TUG and SPPB, such as standing and sitting from a chair. They may also increase gait function by increasing lower extremity strength that is needed to initiate gait, maintain stability while walking, and coordinate limbs. Many of these proposed mechanisms for BFR, though, have been explored in younger adults, and more research is needed to understand these mechanisms in older adults.

To the authors’ knowledge, this is the first systematic review and meta-analysis to examine the effect of BFR on gait and mobility in older adults. Our results support another systematic review that examined BFR on fall risk factors in older adults, including balance performance, TUG, and leg strength [43]. Our review included three additional studies on the TUG and support findings suggesting TUG improvements from BFR. Our review also includes additional walking assessments to examine gait speed. Another meta-analysis combined the TUG and 30 s chair stand to examine physical performance and found significant improvements in the BFR group [44]. Our findings are similar as we found improvements in the TUG and SPPB with the BFR group. While BFR research has largely focused on muscle strength and hypertrophy [16,19], findings from our meta-analysis along with other reviews suggest that BFR may improve mobility and physical function.

For older adults who may not be able to tolerate high-intensity activities, physical therapists or other clinicians may consider combining BFR with low-load resistance training or walking to improve mobility and physical functioning. For older adults at a higher risk for falls, BFR may serve as an alternative tool to potentially prevent future falls. To better understand how BFR affects gait, however, future studies should include walking tasks over shorter distances (e.g., 25-foot walk test) or with everyday activities, such as walking up and down stairs or crossing over obstacles. Future studies should also examine additional gait measures, such as gait variability, stride length, and step width, during both overground walking and real-world walking to better understand which aspects of gait are most affected by BFR. Studies included in this meta-analysis utilized BFR from 24 to 48 sessions, and while the dose–response is unclear, ranges from 6 to 16 weeks of BFR with 3–5 sessions per week appears to improve TUG or SPPB performance. Future studies should also determine the most effective mode of BFR (i.e., walking, resistance training) and the frequency to improve gait performance. Understanding how BFR can be most effectively utilized to improve gait in older adults can help clinicians tailor their treatment plans.

Strengths of this systematic review and meta-analysis include a moderate-to-high quality assessment of studies and low-to-unclear risk of bias. Future studies should report adherence rates and power analyses to improve quality assessment. The number of studies, however, included in this review is small, and there was large heterogeneity across studies examining BFR on gait speed and the SPPB. This variability may result from different types of interventions, frequency and duration of interventions, intensity of exercise sessions, sample sizes, and cuff pressure protocols. With only nine studies included in this systematic review, our results and clinical recommendations to provide BFR for older adults should be interpreted with caution. More studies with larger sample sizes are needed to better determine the effect of BFR on gait outcomes and to help clinicians make BFR recommendations. Another limitation of this study was excluding non-English articles, as this may have excluded studies providing valuable data about BFR and gait outcomes. Future studies from researchers understanding other languages should include non-English articles. Lastly, this review included studies with an average age of 60 years, as two of the studies included some participants under 60. Increasing our age criteria would have further reduced the total number of included studies. Adults 65 years and older, however, may be less tolerable to high-intensity exercise and may benefit more from BFR. Future research, therefore, should focus on recruiting adults 65 years and older.

## 5. Conclusions

In conclusion, this systematic review and meta-analysis provides novel evidence on the effect of BFR on gait performance in older adults. Results indicate that BFR training in comparison to no BFR improved TUG and SPPB performance but not gait speed over 400 m or 6 min. BFR may be more effective for mobility tasks utilizing greater leg strength than endurance-related tasks. There is a need for more studies in this area to better inform clinicians on how to prescribe BFR for older adults to improve gait performance. This review provides evidence from nine studies that BFR may provide an alternative strategy to traditional resistance training for older adults to improve mobility.

## Figures and Tables

**Table 1:** Study characteristics for all included studies. Abbreviations: BFR = blood flow restriction; TUG = Timed Up and Go; 6MW = 6-Minute Walk Test; SBP = systolic blood pressure; DBP = diastolic blood pressure.

Study	Sample Size	Age Mean (SD)	Intervention and Control Group	BFR Cuff Pressure	Frequency	Gait Outcomes	Main Findings
Abe et al. [28]	19	Between 60 and 78 years	Exp: BFR during 20 min of walking Control: no exercise	160 mmHg that was increased by 10 mmHg each week until 200 mmHg	5×/week 6 weeks	TUG	TUG improved in BFR group. No changes in control group.
Clarkson et al. [29]	19	BFR: 69 (6) Con: 70 (7)	Exp: BFR during walking program (10 min) Con: walking program (10 min)	60% of limb occlusion pressure	4×/week 6 weeks	TUG 6MWT	TUG and 6MWT improved in BFR group.
Cook et al. [30]	36	75.6 (7.8)	Exp 1: low-load (30% 1 RM) resistance training with BFR (3 sets of 10 reps) Exp 2: high-load (70% 1 RM) resistance training (3 sets of 10 reps) Con: light upper body resistance and flexibility training	1.5× brachial systolic blood pressure	2×/week 12 weeks	400 m walking speed SPPB	Walking speed improved for all groups.
Harper et al. [31]	35	BFR: 67.2 (5.2) Con: 69.1 (7.1)	Exp: 20% 1 RM resistance training with BFR Con: 60% 1 RM resistance training	0.5*SBP + 2*thigh circumference + 5	3×/week 12 weeks	400 m walking speed SPPB	No significant differences in gait speed or SPPB between groups.
Kargaran et al. [32]	24	62.9 (3.1)	Exp 1: dual-task walking with BFR Exp 2: dual-task walking program Con: continue everyday activities	0.893*thigh circumference +0.734 (DBP) + 0.912(SBP) − 220.046	3×/week 8 weeks	TUG 6MWT	TUG time and gait speed improved in both dual-task groups but more in the BFR + dual-task group.
Letieri et al. [14]	23	69.4 (5.73)	Exp: low-intensity exercise with BFR (40–50 min) Con: no change in activity	80% of total blood flow interruption pressure determined by a vascular Doppler	3×/week 16 weeks	TUG 6MWT	BFR improved walking distance and decreased TUG time.
Ozaki et al. [17]	18	BFR: 64 (1) CON: 68 (1)	Exp: 20 min walking at 45% HRR with BFR Con: 20 min walking at 45% HRR	140 mmHg was increased by 10 mmHg each week until 200 mmHg	4×/week 10 weeks	TUG	TUG improved in BFR group.
Pereria et al. [33]	24	63.1 (5.2)	Exp 1: 4 sets of 15 reps at 30% 1 RM with BFR Exp 2: 3 sets of 10 reps at 70% 1 RM Con: no exercise	50% of restrictive pressure	2×/week 16 weeks	TUG	No difference between BFR and high-load resistance; both saw improvements compared to control in TUG time.
Yokokawa et al. [34]	51	BFR: 72.3 (4.5) Con: 71.0 (4.1)	Exp: low-intensity training with BFR Con: dynamic balance exercises	between 70 mmHg and 1.2× SBP	1×/week 8 weeks	TUG 10 m walk test	BFR improved TUG time. No significant differences in gait speed between groups.

**Table 2:** Study quality assessment for all studies. Abbreviations: Y = yes; N = no; CD = cannot determine.

Study	Research Question	Inclusion/Exclusion Criteria	Randomization	Main Findings	Groups Balanced at Baseline	Outcome Measures Well Defined	Valid and Reliable Measures	Appropriate Protocol	Adherence	Drop-Out	Power Analysis	Confounders	Overall Quality
Abe et al. [28]	Y	Y	Y	Y	Y	Y	Y	Y	CD	Y	N	CD	Good
Clarkson et al. [29]	Y	Y	Y	Y	Y	Y	Y	Y	CD	Y	N	N	Good
Cook et al. [30]	Y	Y	Y	Y	Y	Y	Y	Y	Y	Y	Y	CD	Good
Harper et al. [31]	Y	Y	Y	Y	Y	Y	Y	Y	N	Y	N	N	Good
Kargaran et al. [32]	Y	Y	Y	Y	Y	Y	Y	Y	Y	Y	N	N	Good
Letieri et al. [14]	Y	Y	Y	Y	Y	Y	Y	Y	CD	Y	N	N	Good
Ozaki et al. [17]	Y	Y	Y	Y	Y	Y	Y	Y	CD	CD	N	N	Fair
Pereria et al. [33]	Y	N	Y	Y	Y	Y	Y	Y	CD	N	Y	N	Fair
Yokokawa et al. [34]	Y	Y	Y	Y	Y	Y	Y	Y	Y	N	N	N	Good

**Table 3:** Risk of bias assessment for all included studies as determined using the Cochrane Risk of Bias guidelines. The symbol + represents low risk of bias; ? unclear risk of bias; − high risk of bias.

	Random Sequence Generation (Selection Bias)	Allocation Concealment (Selection Bias)	Blinding of Participants and Personnel (Performance Bias)	Blinding out Outcome Assessment (Detection Bias)	Incomplete Outcome Data (Attrition Bias)	Selective Reporting (Reporting Bias)	Other Bias
Abe et al. [28]	?	?	?	?	+	?	+
Clarkson et al. [29]	+	+	?	?	+	?	+
Cook et al. [30]	+	?	?	?	+	?	+
Harper et al. [31]	+	+	+	+	+	+	+
Kargaran et al. [32]	?	?	+	+	?	?	+
Letieri et al. [14]	+	+	+	+	+	+	+
Ozaki et al. [17]	+	+	?	?	?	?	+
Pereria et al. [33]	+	+	?	?	+	+	+
Yokokawa et al. [34]	+	+	?	?	+	+	+

## Data Availability

The data supporting the conclusions of this article will be made available by the authors on request.
